# Innovative strategy for extraction of green cardamom via super critical fluid extractor at different levels of pressure with its application against microorganisms in vitro and in silico

**DOI:** 10.1186/s40643-025-00951-z

**Published:** 2025-12-08

**Authors:** Sulaiman A. Alsalamah, Mohammed Ibrahim Alghonaim, Khatib Sayeed Ismail, Abdullah Mashraqi, Tarek M. Abdelghany

**Affiliations:** 1 Department of Biology, College of Science, Imam Mohammad Ibn Saud Islamic University (IMSIU), 11623 Riyadh, Saudi Arabia; 2 Department of Biology, College of Science, Jazan University, 45142 Jazan, Saudi Arabia; 3 Botany and Microbiology Department, Faculty of Science, Al-Azhar University, Cairo, 11725 Egypt

**Keywords:** Super critical fluid extractor, Cardamom, Antimicrobial, Antihemolytic, Ultrastructure, Docking

## Abstract

The extraction of bioactive compounds from plants has emerged as a promising strategy for developing resource-efficient solutions that are both economically viable and value-driven. Supercritical fluid extraction (SFE), has become a popular technique for extraction significant plant-based compounds. Our investigation contrasted the yield, biological functions and phytochemical compositions of green cardamom extracts generated with SFE at 100, 200, and 300 bar of pressures. The maximal obtained weight was 0.279 gm upon applying 300 bar. There is a proportional elevation in the levels of most of phenolic compounds which detected using HPLC upon raising the pressure levels for extraction. Gallic acid had a significant increase (*P* ≤ 0.05) upon applying ascending pressure levels The extract obtained at 300 bar demonstrated the most potent antimicrobial activity against *Bacillus subtilis* and *Candida albicans*, with inhibition zones of 23.33 ± 0.58 mm and 22.17 ± 1.04 mm, respectively. Furthermore, antibiofilm and anti-hemolytic assays confirmed that higher extraction pressure enhanced the bioactivity of the extracts, with 300 bar showing the maximum effect. Time-kill kinetics demonstrated a progressive increase in microbial inhibition over time, with the 300-bar extract again displaying the most effective results. Transmission electron microscopy (TEM) revealed significant ultrastructural damage in *B. subtilis* and *C. albicans* treated with the 300-bar extract, indicating strong antimicrobial action at the cellular level. The molecular docking performance of the main constituents in green cardamom extracts gallic acid and syringic acid against *B. subtilis* (PDB ID: 5VX6) and *S. aureus* (PDB ID: 3V8J) using the molecular operating environment (MOE) software was evaluated. The docking scores (S), root mean square deviation (RMSD)_refine values, and energy terms (E_conf, E_place, E_score1, E_refine, E_score2) were analyzed to assess binding affinities. Key interactions, including hydrogen bonds, were identified, with distances and energies quantified. Syringic acid exhibited better binding (S = − 4.27 to − 5.04 kcal/mol) compared to gallic acid (S = − 4.11 to − 4.68 kcal/mol) across both targets. Interactions with residues like GLU 187 (Glutamic acid residue at position 187 in the protein sequence) and ARG 172 (Arginine residue at position 172) in 5VX6, and ASP 239 (ASP 239: Aspartic acid residue at position 239) in 3V8J, highlighted critical binding motifs. The findings concluded that green cardamom extracts, particularly those obtained at 300 bar, possess enhanced antimicrobial and antibiofilm properties, supported by both experimental and computational evidence. These results highlight the therapeutic potential of pressure-optimized SFE in maximizing the bioactivity of plant extracts.

**Graphical abstract:**

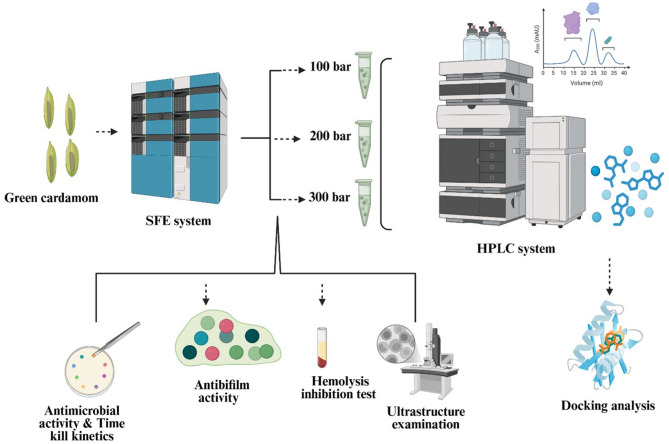

## Introduction

The academic sector has long been interested in aromatic and therapeutic plants with antimicrobial capabilities because they provide numerous biologically active substances that can be applied to counteract the spreading of microorganisms that cause serious and potentially deadly infections (Abdelghany et al. 2020; Alawlaqi et al. 2023). In Mediterranean areas, spices and herbaceous plants are commonly used in preparing food since they have the ability to fight towards several pathological bacteria (Al-Rajhi et al. 2025a). The frequent consumption of antimicrobial agents among consumers to combat pathogens explains the rise in resistance produced in most populations towards microbial infections which urge the need to use innovative tools to overcome these infections (Abdelghany et al. 2019; Abdullah et al. 2022).

The green cardamom species (*Elettaria cardamomum)* is a big evergreen herb rhizomatous monocot species that is a member of the Zingiberaceae and Elettaria families (Castillo et al. 2023). It is widely recognized for the flavorful components and bioactive substances found in dry mature fruit, that make them valuable commodities for the food, medicinal products and cosmetic sectors (Teresa-Martínez et al. 2022). In the food industry, cardamom is frequently used to flavor prepared meals, soft drinks, and confections. Furthermore, it is employed in the cosmetics industry for body and hair care items, aromatherapy, and scents (Guzmán and Lucia 2021). In the drug sector, cardamom has also been utilized as a potent antioxidant, antimicrobial agent (Ashokkumar et al. 2020).

Traditionally, extraction of components and bioactive substances has relied on conventional methods such as solvent extraction or steam distillation. However, these methods often present challenges including solvent residue, thermal degradation, and limited extraction efficiency (Al-Rajhi et al. 2025b; Alsalamah et al. 2025). In recent years, Supercritical Fluid Extraction (SFE) has emerged as an advanced and environmentally friendly technique capable of improving yield and preserving sensitive bioactive compounds through the use of supercritical CO_2_. In this regard, supercritical extraction methods have become a crucial path, with their long history that reflects the development of extraction techniques. Supercritical CO_2_ extraction is thought to be a more ecologically friendly and efficient process than solvent-based extractions as it has a positive value including its non-flammability, non-toxicity, and ease of recycling (Almehayawi et al. 2024; Bazaid et al. 2025). Although several studies have applied SFE for extracting natural constituents from various plant materials, the effect of different pressure levels during SFE on the yield, chemical composition, and biological activity of *E. cardamomum* remains underexplored.

The bioactive components are being characterized and quantified using a variety of methods involving chromatography following the extraction of phytochemicals. Cardamom phytochemicals have been shown to offer several health benefits in recent years, including antioxidative, antibacterial, and anti-hypertensive effects (Chakraborty et al. 2019; Yousefi et al. 2019). In rats fed a high-fat diet, cardamom phytochemicals dramatically reduced the levels of triglycerides (Nagashree et al. 2017). Cardamom phytochemicals were shown to suppress the growth of bacteria and fungi that cause foodborne illnesses (Abdullah et al. 2021).

The reactive nature of free radicals can be inhibited by antioxidant substances (Nurcholis et al. 2022). An inherent means of defense versus reactive oxygen species (ROS) is the production of indigenous or intracellular antioxidants by the human body. It was discovered that plant-based antioxidants were more successful than chemical-based ones in reducing ROS levels (Neha et al. 2019). Secondary plant metabolites involve molecules known as polyphenols, which include phenolic and flavonoid chemicals, are molecules that contain several phenolic rings with several useful roles (Al-Rajhi et al. 2023a; Selim et al. 2024). A useful technique that simplifies the conduct of in vitro investigations by doing away with the need for high-throughput testing of a large number of molecules commonly used in the drug development process is molecular docking investigation (Al-Rajhi et al. 2022, 2023b, c; Yahya et al. 2022). There are historical studies used SFE to extract cardamom (Gopalakrishnan and Narayanan 1991; Hamdan et al. 2008). There is a great need to optimize different method of extraction of natural products to use these extracts in various biomedical applications (Dzobo 2022).

There may be a risk of contamination of many food products by *Bacillus subtilis* (*B. subtilis*). To stop and manage the emergence of *B. subtilis*, cleaning procedures and the application of biological substances are advised in the targeted danger regions (Cho and Chung 2017). Essential oils and compounds produced from plants are among the natural items that have demonstrated efficacy against *Staphylococcus aureus* (*S. aureus*). These organic substances have the ability to stop the development of bacteria, break up biofilms, and alter virulence factors such the formation of hemolysin (Kashi et al. 2024). Significant difficulties in treating *Klebsiella pneumoniae* (*K. pneumoniae*) clones underscore the need for novel, more potent pathogen control strategies. We don’t fully grasp the processes underlying acquired or natural antibiotic resistance in *K. pneumoniae*. Thus, it is essential to keep looking for new compounds to combat *K. pneumoniae* and to completely comprehend the mechanisms underlying its antibiotic resistance (Huy 2024). According to recent research, cardamom essential oil may prevent the synthesis of violacein, which in turn prevents bacteria like *Salmonella typhi* (*S. typhi*) from communicating and forming biofilms through a process known as bacterial quorum sensing (Abdullah et al. 2021). Several natural products exhibit antifungal properties against *Candida albicans* (*C. albicans* and *Penicillium glabrum* (*P. glabrum*) (El-Baz et al. 2021; Cabañas et al. 2020). Many investigators use various classical extraction methods for production of cardamom extract and test its antimicrobial activity (Noumi et al. 2018a, b; Sobhy et al. 2025; Pourkhosravani et al. 2021; Hleba et al. 2024; Sulieman et al. 2023). Moreover, while the antimicrobial properties of cardamom extracts have been studied in vitro, and due to the development of resistant microbes more in vitro and in silico investigations that link the chemical profile of extracts with their potential molecular interactions against microbial targets (Noumi et al. 2018a, b; Sobhy et al. 2025; Pourkhosravani et al. 2021; Hleba et al. 2024; Sulieman et al. 2023; Gupta et al. 2023). This gap hinders a comprehensive understanding of the mechanism of action and the optimization of extraction parameters for therapeutic applications. Therefore, the present novel work was aimed to introduces an innovative approach by evaluating green cardamom extracts obtained at various SFE pressures levels (100, 200, and 300 bar), it is different from the previous studies as it is characterizing extracts phytochemical profiles using HPLC, and assessing their antimicrobial activity through both in vitro assays and molecular docking simulations.

## Materials and methods

### Plant & chemicals

Green cardamom powder was purchased from an Egyptian market authorized retailer (Giza, Egypt Code: 03547). The container of plastic was used to hold the powder at the ambient temperature for further experimentation. The used chemicals were purchased from (MOPOCO Co. INC., Egypt).

### SFE condition for green cardamom

In a supercritical fluid extraction device (Waters, USA), five grams of green cardamom powder are processed. A high-pressure piston forced the carbon dioxide of the cylindrical vessel onto the chiller and to the extraction tank. The instrument panel of the extraction device was set to the extract’s length (15 min for statistics, and 45 min for dynamic extraction), the temperature at which it operated (36 °C), and the pressures of 100 (Code: 1), 200 (Code: 2), and 300 bar (Code: 3). The extraction device unsealed the fitting across the pump and the specimen cartridge, enabling 5.0 mL/min of CO_2_ to flow across the specimen once the required temperature and pressure were reached. Following each the extraction procedure, the experiment has repeated three times the weights were determined and the extract was taken away in a glass container (Selim et al. 2025).

### HPLC testing for detecting flavonoids and polyphenols compounds in green cardamom extracts

To quantify and identify the flavonoid and polyphenol content in green cardamom extracts, High-Performance Liquid Chromatography (HPLC) was performed using a (PerkinElmer LC300 system, Germany) equipped with a photodiode array (PDA) detector set to 285 nm for optimal detection of phenolic compounds. Chromatographic separation was carried out using a KNALJER C18 reversed-phase column (4.7 mm × 250.0 mm, 6 μm) maintained at a temperature of 42 °C. Each sample injection volume was 5.0 µL. The mobile phase consisted of: 0.06% trifluoroacetic acid (TFA) in distilled water (Solvent A) and Acetonitrile (Solvent B). In the subsequent control, the mobile phase was progressively altered in a straight gradient from 0 to 6 min (81%), 6 to 9 min (60%), 9 to 13 min (60%), for minutes 13 to 16 (83% 1st), for minutes 16 to 18 (82%), and for minutes 18 to 23 (83%). Polyphenolic compounds were identified by comparing the retention times and UV–Vis spectral data of peaks with those of known standards, including gallic acid, catechin, quercetin, and others. Quantification was conducted using external calibration curves based on standard solutions of reference compounds (Alsalamah et al. 2024).

### Action of various extracts of green cardamom samples towards test microbes

A group of various test food-born microbes including: *Bacillus subtilis* (ATCC6051), *Staphylococcus aureus* (ATCC43300), *Klebsiella pneumoniae* (ATCC2146), *Salmonella typhi* (ATCC14028), *Candida albicans* (ATCC90028), and *Penicillium glabrum* (ATCC11080) were applied as test microbes. The fungi were streaked onto the area of the malt extract culture in the petri dishes, and the examined bacteria were injected onto the surface of the nutrient agar. Wells (5.0 mm) were formed in the infected agar layer, subsequently 100 µL of various extracts of green cardamom were placed in each well. The standard antifungal was fluconazole at 1000 µg/mL, while the standard antibiotic was gentamicin at 20 µg/mL. To allow the studied components to diffuse before microbial development, the plates were stored at 5 °C for 20 min. Following a 24-h incubation period at 37 °C (for bacteria), 5–7 days at 28 °C (for fungi) the inhibition zones surrounding the wells were measured and compared to the inhibition zone to negative control and the inhibition zone of the standard drug (Abdel Ghany and Hakamy 2014).

### Evaluation the values of MIC and MBC of green cardamom samples towards test microbes

The minimal inhibitory levels (MIC) of investigated samples versus microorganisms (*B. subtilis, S. aureus, K. pneumoniae, S. typhi,* and *C. albicans*) were determined using the micro-dilution technique. 100 µL of repetitions of each created level were evaluated in a fluid medium made up for each location in 96 tissue culture plates, while each specimen had been diluted for values ranging from 0.95 to 1000.0 µg/mL. A 1.3 × 10^6^ CFU/ml was obtained by inoculating microbe cultures (1.0 McFarland standard) with 2.9 µl of sanitized 0.9% sodium chloride for every well. The bacteria were cultivated in the wells for 3 days at 38 °C, while the fungal strains were left to grow for 5–8 days at 28 °C. While specimens containing 100% inhibitory agent were moved to the holes for appropriate incubation periods of time, the organisms being studied were cultivated on their appropriate media for minimal bactericidal levels (MBC), which was the lowest level of specimens that could not sustain the growth of examined microbes in the examined circumstance. (Al-Rajhi et al. 2023b).

### Antibiofilm action of prepared specimens of green cardamom

The effect of the tested materials on the growth of bacterial biofilms was evaluated in 96-well plates. To put it succinctly, 300 μL of freshly introduced trypticase soy yeast broth (TSY) was divided into each microplate well, and it was cultured with previously determined sub-lethal concentrations of MBC (75, 50, and 25%). The ultimate amount of TSY was 10^6^ CFU/mL. Wells containing the medium, methanol, and no extracts were used as controls. The plates were then left at 38 °C for 2 days. Following the incubation process the supernatant was discarded, and sterile distilled water was used to thoroughly clean the free-floating bacterial cells from every well in the experiment. After 35 min of air drying, the plates were dyed with a 0.1% crystal violet fluid solution for 15 min at ambient temperature to reveal the biofilm that had formed. After incubation, sterile distilled water was used to remove any remaining stain. Lastly, 250 μL of 95% ethanol was added to each well to dissolve the dye that was bound to the bacterial cells. After 16 min of incubation, absorption was determined at an intensity of 580 nm employing an Elisa reader. The calculation was done as the following: % antibiofilm activity = [(O.D. _control_ − O.D. _sample_) / OD. _control_] * 100. Where, O.D _control_ refers to the optical density of a control group (a well with only bacteria and media) and OD _sample_ represents the optical density of the test group (Slobodníková et al. 2016).

### Hemolysis inhibition test

The hemolysin effects of samples of green cardamom in sub-MIC (25.0%, 50.0%, and 75.0% of MIC) were measured using the Rossignol et al. (2008) technique. After being adjusted to an OD_595_ of 0.4, the tested bacteria underwent spinning at 20,000×*g* for 23 min with 25.0%, 50.0%, and 75.0% of MIC (sub-MIC) or samples that were untreated. 500 µL of supernatants were combined with freshly made erythrocyte solution (2.0%) in 0.80 mL of saline, kept at 36 °C for 120 min, and then spun at 15,000×*g* for ten minutes at 4 °C. To generate a positive control, an erythrocyte suspension was combined with 0.1% sodium dodecyl sulfate. For generating a control set of un-hemolyzed erythrocytes, erythrocytes were cultured in LB broth under similar circumstances. Monitoring was carried out in triplicate, and the amount of hemoglobin produced was determined through assessing absorption at 545 nm. Mean ± standard error of the variation in percentage from the hemolysis of the control group with no treated settings was used to represent the hemolysis that occurred with samples that were tested in sub-MIC. The level (%) of cells hemolysis was determined as following: % = [(a − b)/(t − b)] × 100. Where: a is the absorbance level for the tested extract, b (baseline) is a negative norm related to RBCs left with 600.0 μL sterile media and t is a positive norm related to the total hemolysis resulted by left RBCs in media enriched with 0.10% SDS (final level).

### Killing kinetic times test

This investigation was conducted to investigate the time dying of evaluated for test specimens against the test microorganisms. 0.7 McFarland solution was combined with fresh bacterial stains. Containers containing broth media infected with 1.10 × 106 bacterial strains were combined with the MIC values of the examined oil forms. At 0, 30, 60, 90, 120, 150, and 180 min, 0.5 mL of samples were taken, cultured on dishes, and stored at 38 °C for the night. However, organisms lacking tested samples were present in control sample. Count the number of colonies (colony-forming units, CFU) at each time point. Calculate the reduction in CFU/mL at each time point compared to the initial inoculum. Test Time-Dependent Dependent Effects. Over the course of the test, a shift in the number of bacterial cells was calculated with strict adherence to standardized protocol. Careful analysis of the results is necessary to determine the impact of various pressure levels on antimicrobial’s activity relative to control (standard drug) results (Qureshi et al. 2022).

### Ultrastructure examination using transmission electron microscope

The specimens of *B. subtili*s and *C. albicans*, comprising untreated microbes and those subjected to the MIC of green cardamom (extracted at 300 bar), each, had been fixed in an aqueous glutaraldehyde (2.6%) solution for 48 h at 5 °C, post-fixed with a 1% osmium tetroxide liquid for 4.0 h at 5 °C, sectioned via an ultra-microtome (Leica, Germany), and then variation in the ultrastructure were investigated with a TEM (JEOL 1010) (Nguyen et al. 2021).

### Docking analysis

Molecular docking was performed using MOE (Chemical Computing Group) (Khelfaoui et al. 2021) with the following parameters:

*Protein preparation* Structures 5VX6 and 3V8J were retrieved from the PDB, protonated, and energy-minimized then the site finder created the active binding sites, which served as the binding pocket’s dummy sites.

*Ligand preparation* Gallic acid and syringic acid were optimized with the MMFF94x force field to achieve a low-energy conformation.

*Docking procedure *The ligands were put at the site using the triangle matcher approach, and the stiff receptor atoms were docked for 100 ns. The GBVI/WSA dG processes were used for rescoring, with the London dG acting as a scoring function. For each ligand–protein pair, several postures were created, and the top five were chosen for further investigation. 2D and 3D interaction diagrams were created to show how both ligands attach to each protein’s active regions. These graphical representations focused on specific interactions. The docked complexes were examined to identify the interactions between the studied ligands and the active site residues of the protein.

### Statistics for the results

The findings of the study were verified three times using the mean standard deviation (± SD). The outcome of experiments was evaluated by a one-way ANOVA evaluating three separate groups’ means for the independent variable at various applied pressure levels in each experiment, and ANOVA followed by followed by post-hoc test for to explore significant differences revealed by the ANOVA using the Graph Pad Prism V8 program (CA, USA).

## Result and discussion

In this work, SFE-CO_2_ of green cardamom seeds powder packed in the Extractor cartilage and exposed to three different pressures 100, 200, and 300 bar. It is clear that this approach was effective in yield of extract. As the pressure raised from 100 to 200 to 300 bar the yield of extract gradually increased from 0.189 to 0.217 to 0.279 g, respectively with a non-significant difference (*P* ≥ 0.05) in the net weight of the produced extract (Table 1). SFE has benefits over conventional extraction techniques. Although higher pressure usually improves extraction, selectivity may be impacted (Sobhy et al. 2025). Certain molecules can be extracted selectively while others are left behind by carefully regulating the pressure. According to earlier study on the SFE fundamental terms, higher pressure raised the SFE’s frequency and diffusion, which improved recovery (Khelfaoui et al. 2021). Furthermore, according to a different investigation, higher pressure has essential influence on the use of SFE in medicinal plants recovery (Zhao et al. 2014) which come in the same line with the present results which revealed a maximal weight for the obtained extract which was 0.279 g could be done upon raising he applied pressure in the SFE system.

**Table 1:** SFE-CO_2_ of green cardamom at different pressure levels

Condition code	Temp. ^0^C	Pressure Bar	Extraction static time (min)	Extraction dynamic time (min)	The used Quantity (gm)	Extract quantity (gm)
1	60	100	15	45	5.0	0.189 ± 0.01^a^
2	60	200	15	45	5.0	0.217 ± 0.01^a^
3	60	300	15	45	5.0	0.279 ± 0.02^a^

Data are reported as means ± SD; similar letters above numbers refer to non-significant difference *P* ≥ 0.05

**Fig. 1:**
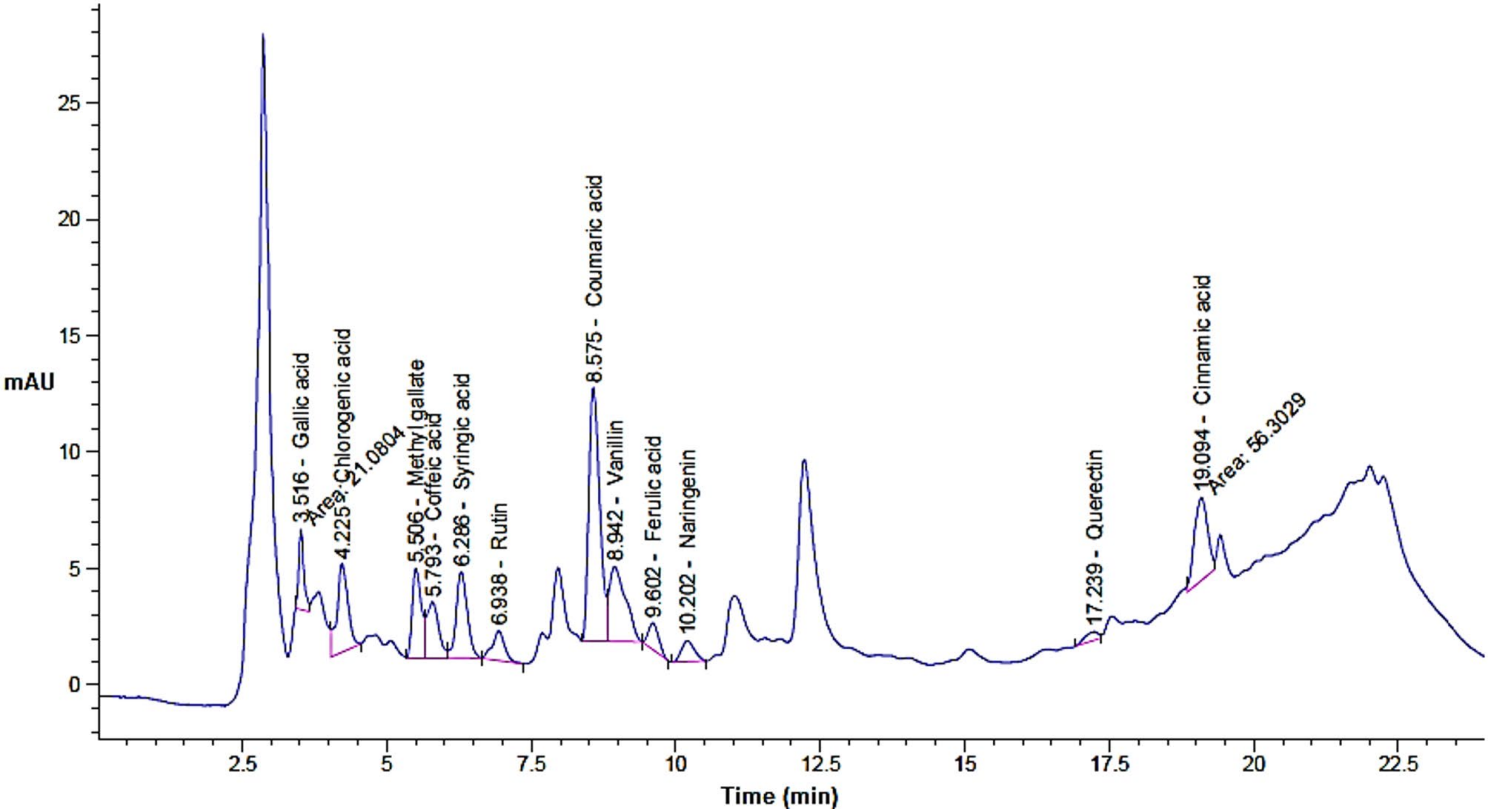
HPLC chromatogram for green cardamom upon SFE extraction at 100 bar

**Fig. 2:**
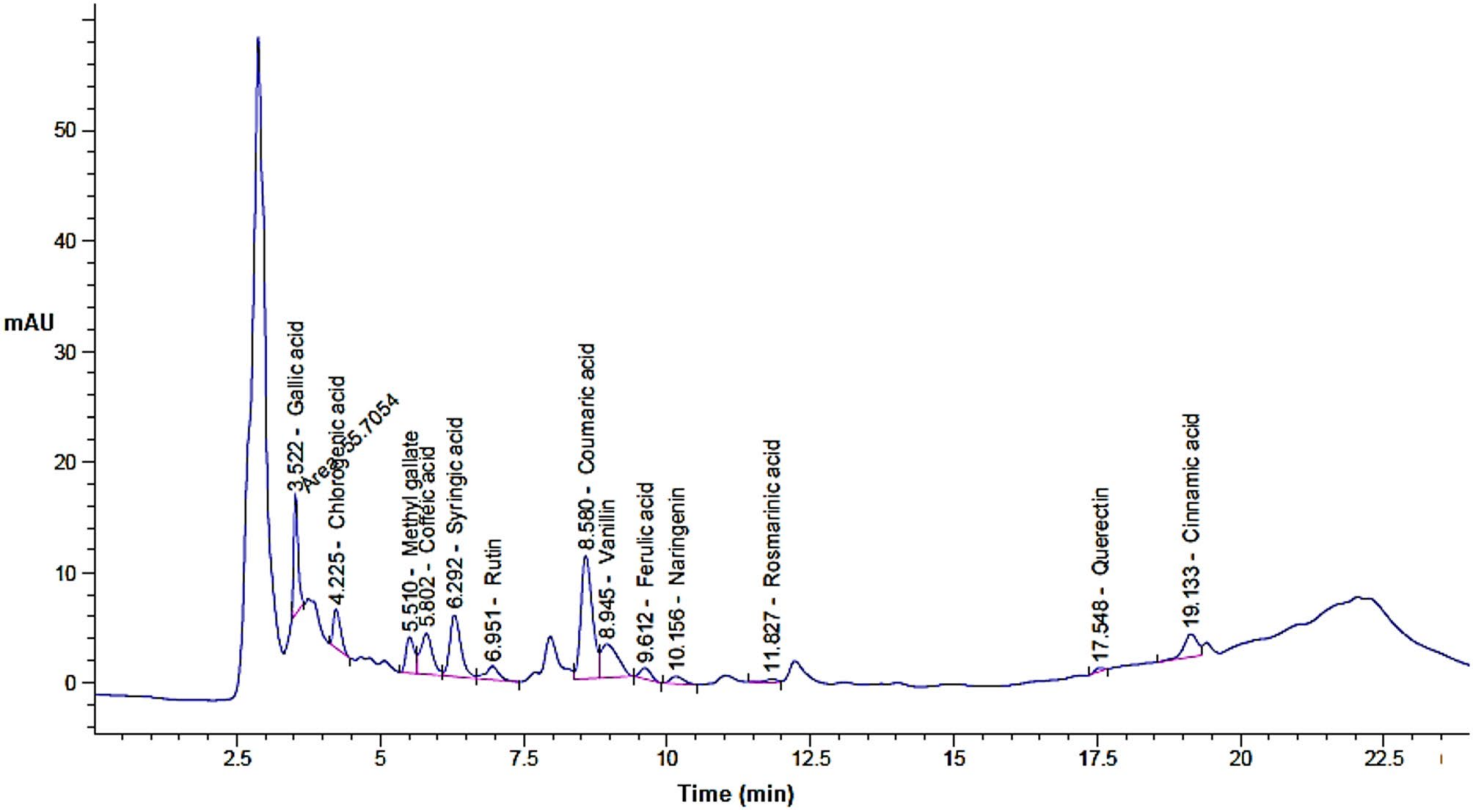
HPLC chromatogram for green cardamom upon SFE extraction at 200 bar

**Fig. 3:**
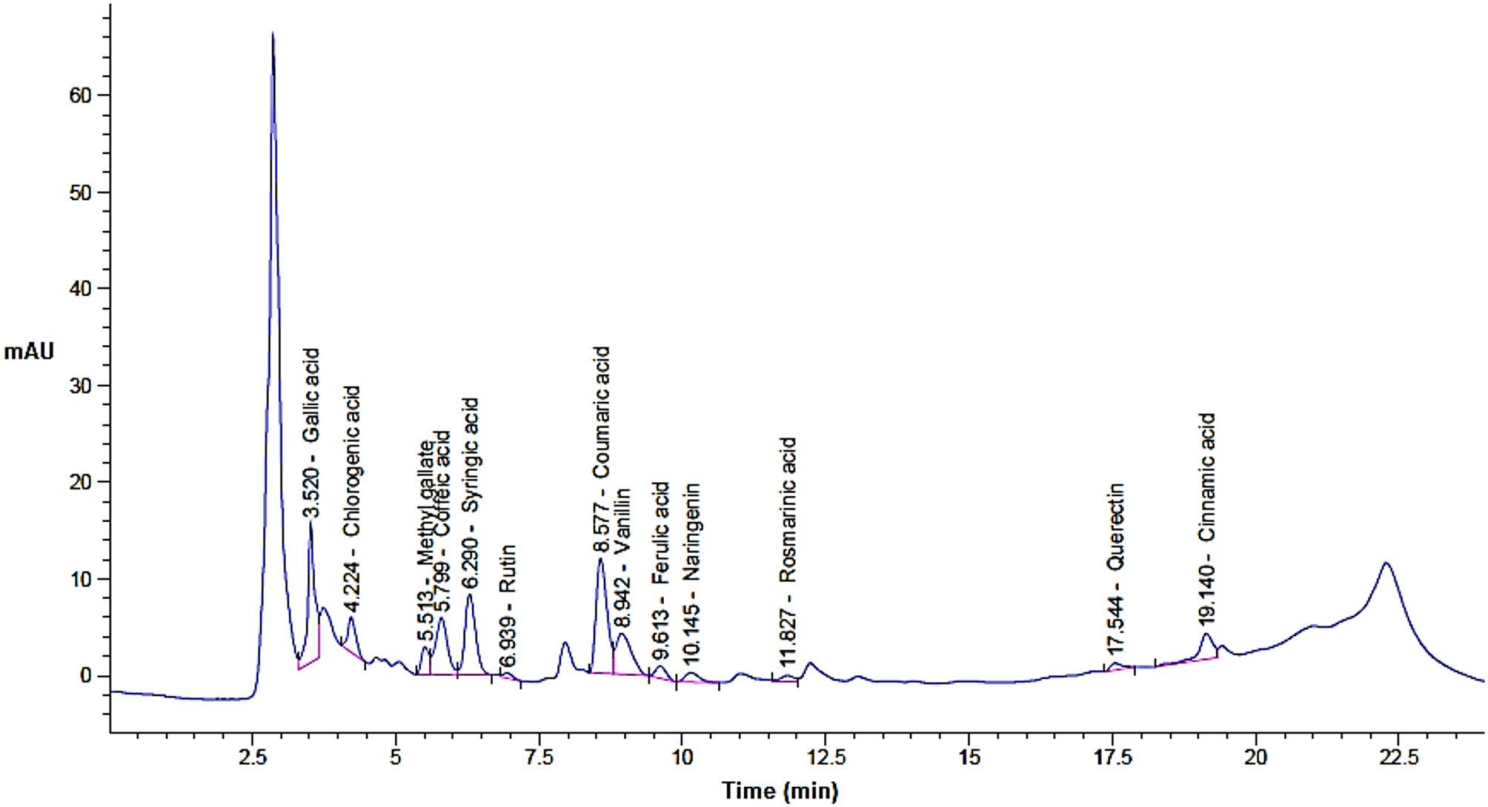
HPLC chromatogram for green cardamom upon SFE extraction at 300 bar

Based on the injected standards of phenols and flavonoids in HPLC, 13 compounds were appeared in the extract but with various quantities depending on the extraction pressure (Table 2 and Figs. 1, 2 and 3). The concentration of eight compounds was increased with the increasing of extraction pressure while the concentration of four compounds was decreased with the increasing of extraction pressure. In SFE, particularly when using supercritical CO_2_, the pressure directly effects the solvent power and selectivity of the extraction process. As pressure increases, supercritical CO_2_ becomes denser and can better dissolve and extract heavier or less polar constituents that were not easily extractable at lower pressure. Also, may be high pressure allows CO_2_ to penetrate deeper into the cardamom extract, liberating more bound phenolics and flavonoids. One compound namely rosmarinic acid was appeared in the extract at extraction condition of 200 and 300 bar with concentration 24.84 and 57.29 µg/g, respectively but didn’t appear in the extract at extraction condition of 100. For instance, gallic acid dramatically increased from 77.17 to 203.93 (*P* ≤ 0.05) to 461.52 µg/g (*P* ≤ 0.01), caffeic acid increased from 87.87 to 137.81 (*P* ≥ 0.05) to 216.83 (*P* ≤ 0.05) µg/g, syringic acid increased from 144.90 to 220.52 (*P* ≥ 0.05) to 315.57µg/g (*P* ≤ 0.05), while chlorogenic acid decreased from 345.20 to 216.64 (*P* ≥ 0.05) to 242.36 µg/g (*P* ≥ 0.05), Rutin shifted from 139.98 to 144.98 (*P* ≥ 0.05) to 45.00 µg/g (*P* ≤ 0.05), methyl gallate decreased from 115.83 to 92.51(*P* ≥ 0.05) to 74.64 µg/g (*P* ≥ 0.05) when the extraction pressure increased from100 to 200 to 300, respectively. HPLC results is a highly efficient and provides analytical quantitively separation as it is mainly used for the phenolics flavonoids in different prepared extracts (Mizzi et al. 2020). Our findings indicated that pressure was effective for releasing the compounds from the extract. Moreover, the obtained results were matching with other investigation studied the SFE at different pressures. The maceration technique (Haido et al. 2024), extraction through supercritical fluid (Ahmed et al. 2022), and the use of Soxhlet extraction (Abdullah et al. 2017) are the techniques most commonly employed for obtaining flavonoid and phenolic components from spices. The main determinants of the quantity of antioxidants obtained are the extraction process and time (Alqahtani et al. 2024). In addition to pressure, other factors that can impact the yields obtained from extraction of molecules, which can be measured as total flavonoid and phenolic contents, include length of time and temperature (Upadhya et al. 2015). Thus, application of SFE at different pressure levels resulted in alteration of the levels of the detected phenolic compounds and flavonoids where certain compounds could be seen at highest pressure level relative to the other applied pressure which in the same line with other reported results by other investigators (Ahmed et al. 2022).

**Table 2:** Phenols and flavonoid compounds of in the extract of green cardamom at different pressure levels

Detected* constituents	100 bar			200 bar			300 bar		
	RT	Area %	Conc. (µg/g)	RT	Area %	Conc. (µg/ml)	RT	Area %	Conc. (µg/g)
Gallic acid	3.52	4.13	77.17^a^	3.52	10.07	203.93^b^	3.52	17.48	461.52^c^
Chlorogenic acid	4.23	9.71	345.20^a^	4.23	5.62	216.64^a^	4.22	4.82	242.36^a^
Methyl gallate	5.51	8.12	115.83^a^	5.51	5.98	92.51^a^	5.51	3.70	74.64^a^
Caffeic acid	5.79	6.72	87.87^a^	5.80	9.71	137.81^a^	5.80	11.72	216.83^s^
Syringic acid	6.29	9.66	144.90^a^	6.29	13.55	220.52^a^	6.29	14.88	315.57^s^
Rutin	6.94	3.67	139.98^a^	6.95	3.50	144.98^a^	6.94	0.83	45.00^y^
Coumaric acid	8.58	27.76	254.42^a^	8.58	28.06	278.96^a^	8.58	21.73	281.54^a^
Vanillin	8.94	11.18	112.15^a^	8.95	12.40	114.72^a^	8.94	10.85	141.94^a^
Ferulic acid	9.60	2.40	38.56^a^	9.61	2.97	41.27^a^	9.61	2.21	46.24^a^
Naringenin	10.20	1.93	49.22^a^	10.16	2.78	65.45^a^	10.15	2.33	77.62^a^
Rosmarinic acid	11.74	0.00	0.00^a^	11.83	0.92	24.84^b^	11.83	1.64	57.29^b^
Quercetin	17.24	0.67	23.06^a^	17.55	1.22	38.72^a^	17.54	1.42	63.83^b^
Cinnamic acid	19.10	11.04	54.56^a^	19.13	6.40	34.31^a^	19.14	6.37	44.52^a^

*Application of various pressure levels resulted in alteration in the concertations of cdetected compounds and different letters refer to significant differnce where a:b *P* ≤ 0.05; b: c *P* ≤ 0.05; a: c *P* ≤ 0.01; a: s *P* ≤ 0.05 (significant increase in case of); a: y *P* ≤ 0.05 ( signifiant decrease in case of rutin); similar letters refer to non-significant differnce *P* ≥ 0.05)

**Fig. 4:**
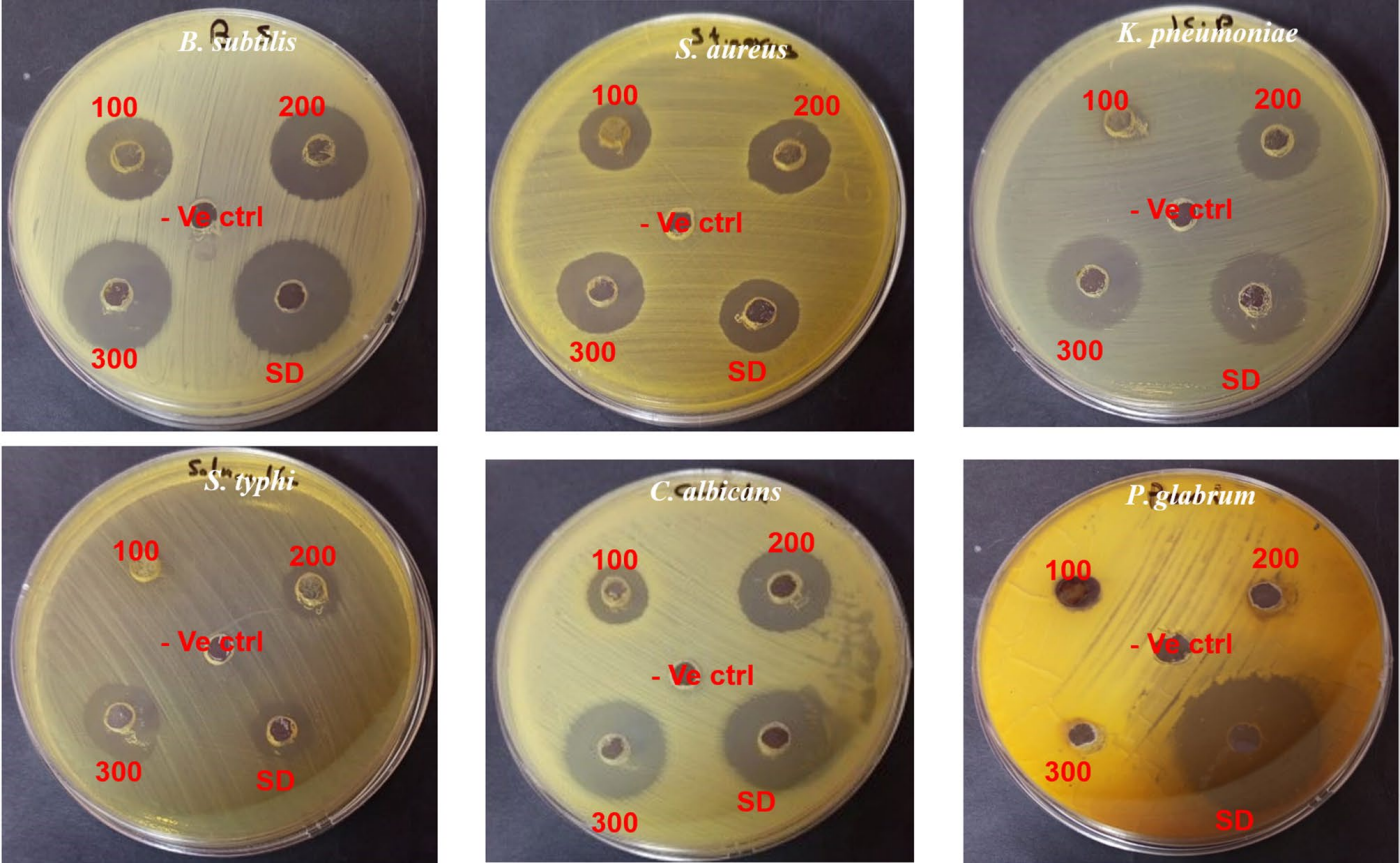
Antimicrobail action of of green cardamom extracts upon using various pressure levels (100, 200, and 300 bar) (-ve: negative control, SD: standard drug)

The recorded data in Table 3 and Fig. 4 showed that the obtained extract at 100, 200, and 300 possess antimicrobial activity against *B. subtilis, S. aureus, K. pneumonia,* and *C. albicans* with various inhibition zones based on the extraction condition and tested microbe. The inhibition zones were 18.17 ± 0.29**,** 20.83 ± 0.29**,** and 23.33 ± 0.58 mm for *B. subtilis*, 8.67 ± 0.58, 15.83 ± 1.04, and 20.33 ± 0.58 mm for *K. pneumonia,* 14.33 ± 0.58, 19.33 ± 2.08, and 22.17 ± 1.04 mm for *C. albicans* employing the extract at 100, 200, and 300 bar, respectively. On the other hand the extract at 100 of the extraction condition faild to inhibit *S. typhi* while exhibited 10.75 ± 0.43 and 16.33 ± 0.58 mm inhibition zones at 200, and 300 of extraction condition. The filamentous fungus *P. glabrum* didn’t inhibited by the extract at any studied extraction condition. The recorded results were compared to the inhibitory action of standard antibiotic/ antifungal drugs. Furthermore, the measured MIC and MBC confirmed that extract efficacy increased with increasing extraction condition of pressure, where less MIC and MBC quantities at 300 bar were recorded than that recorded at 100 or 200 bar against tested microbes. The present results revealed that the prepared extracts had a microbicidal impact where its MBC/MIC levels where calculated ≤ 4. Cardamom seeds add a sweet and tangy flavor. The present results revealed that the antimicrobial properties can be greatly influenced by phenolic and flavonoid content, which have been found in many extracts and have demonstrated the capacity to suppress the development and activity of a variety of microorganisms, including fungus and bacteria. The composition and content of the phenolic and flavonoid molecules can affect the precise antibacterial actions and processes (Abdullah et al. 2017; Alqahtani et al. 2024; Upadhya et al. 2015; Kaderides et al. 2019; Ispiryan et al. 2024). The antimicrobial properties of phenolic and flavonoid compounds can be influenced by their particular chemical structure, which includes the quantity and location of hydroxyl groups (Gumisiriza et al. 2024; Kakouri et al. 2023). The antibacterial characteristics of these chemicals are also significantly influenced by their level of concentration. The sensitivity of various bacterial and fungal species and strains to phenolic and flavonoid substances might vary. The present result in the same line with other investigators who reported that many phenolic and flavonoid compounds have strong antibacterial qualities against all three, although some may be less effective against Gram-negative fungi and bacteria than Gram-positive bacteria (Veiko et al. 2023; Makade et al. 2024; Cucu et al. 2024).Furthermore, an antimicrobial substance that treats dental cavities, it is frequently used as a breath freshener to preserve oral health (Hmidani et al. 2021). Numerous investigations on the extraction of naturally occurring non-antibiotic compounds with possible antibacterial effects—like SFE extracts—for the treatment of multidrug-resistant microbes have been published (Stebuliauskaitė et al. 2025; Appiah et al. 2017). According to reports, an antimicrobial substance is considered both bactericidal and inhibitory if its MBC/MIC ratio is low, often at or below 4:1. While managing severe illnesses when a high level of bacterial clearance is required, this is highly beneficial (Makade et al. 2024).

**Table 3:** Antimicrobail action (mm) & MIC and MBC (µg/ml) levels of green cardamom extracts upon using various pressure levels (100, 200, and 300 bar) (outcomes are represnted as means ± SD)

Tested microbes	Zone of inhibition (mm)				MIC/MBC (µg/ml)*		
	100	200	300	standard	100	200	300
*B. subtilis*	18.17 ± 0.29	20.83 ± 0.29	23.33 ± 0.58	25.17 ± 1.26	125/500	31.25/62.5	15.62/15.62
*S. aureus*	15.50 ± 0.87	17.83 ± 0.29	18.67 ± 1.53	16.33 ± 0.58	125/250	62.5/62.5	31.25/31.25
*K. pneumoniae*	8.67 ± 0.58	15.83 ± 1.04	20.33 ± 0.58	18.67 ± 1.53	1000/500	125/250	15.62/31.25
*S. typhi*	NA	10.75 ± 0.43	16.33 ± 0.58	14.67 ± 1.15	–	250/500	250/500
*C. albicans*	14.33 ± 0.58	19.33 ± 2.08	22.17 ± 1.04	21.83 ± 0.29	125/250	62.5/125	31.25/62.5
*P. glabrum*	NA	NA	NA	28.33 ± 2.52	–	–	–

*Calculation of MIC/MBC values refer to a microbicidal roles of examined samples towards test organisms where the resulted values where ≤ 4

**Fig. 5:**
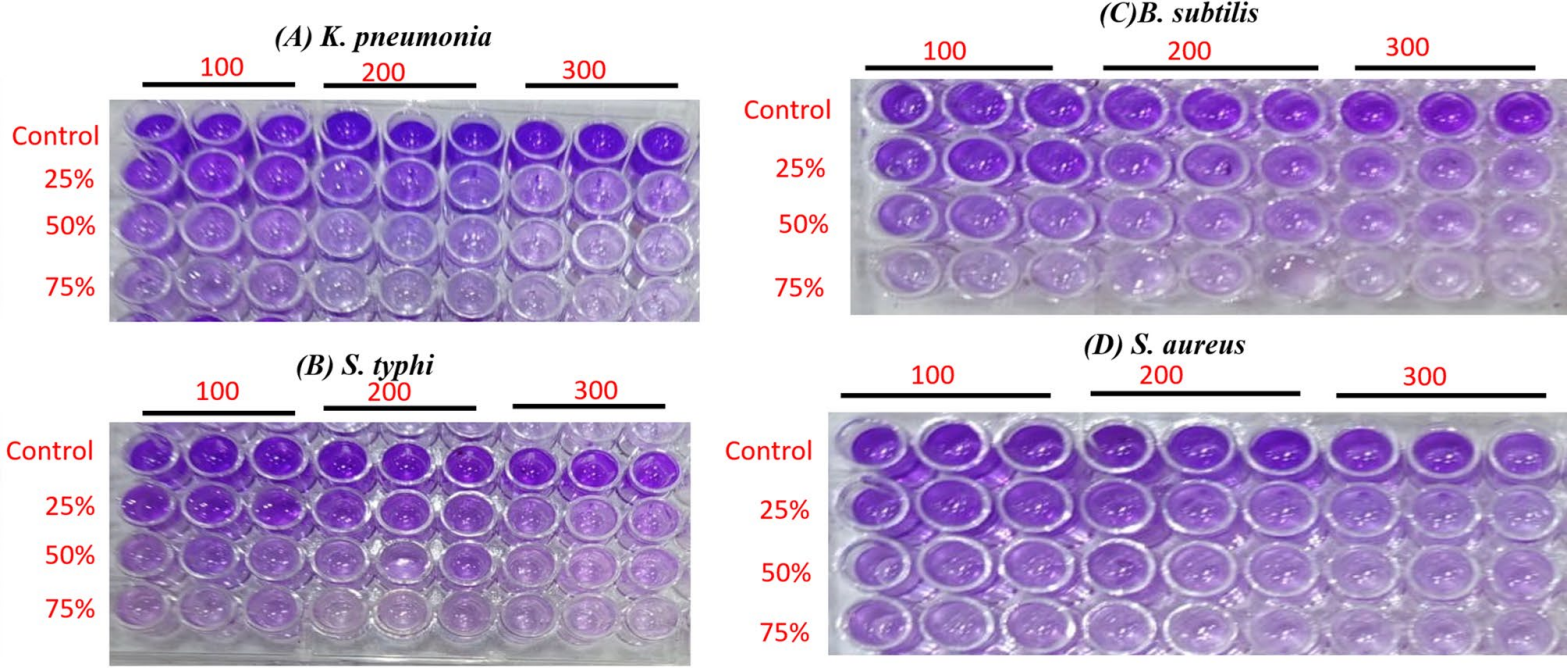
Antibiofilm action of green cardamom extracts upon using various pressure levels (100, 200, and 300 bar) towards **A*** K. pneumonia,* **B** *S. typhi,* **C** *B. subtilis* and **D** *S. aureus*

**Fig. 6:**
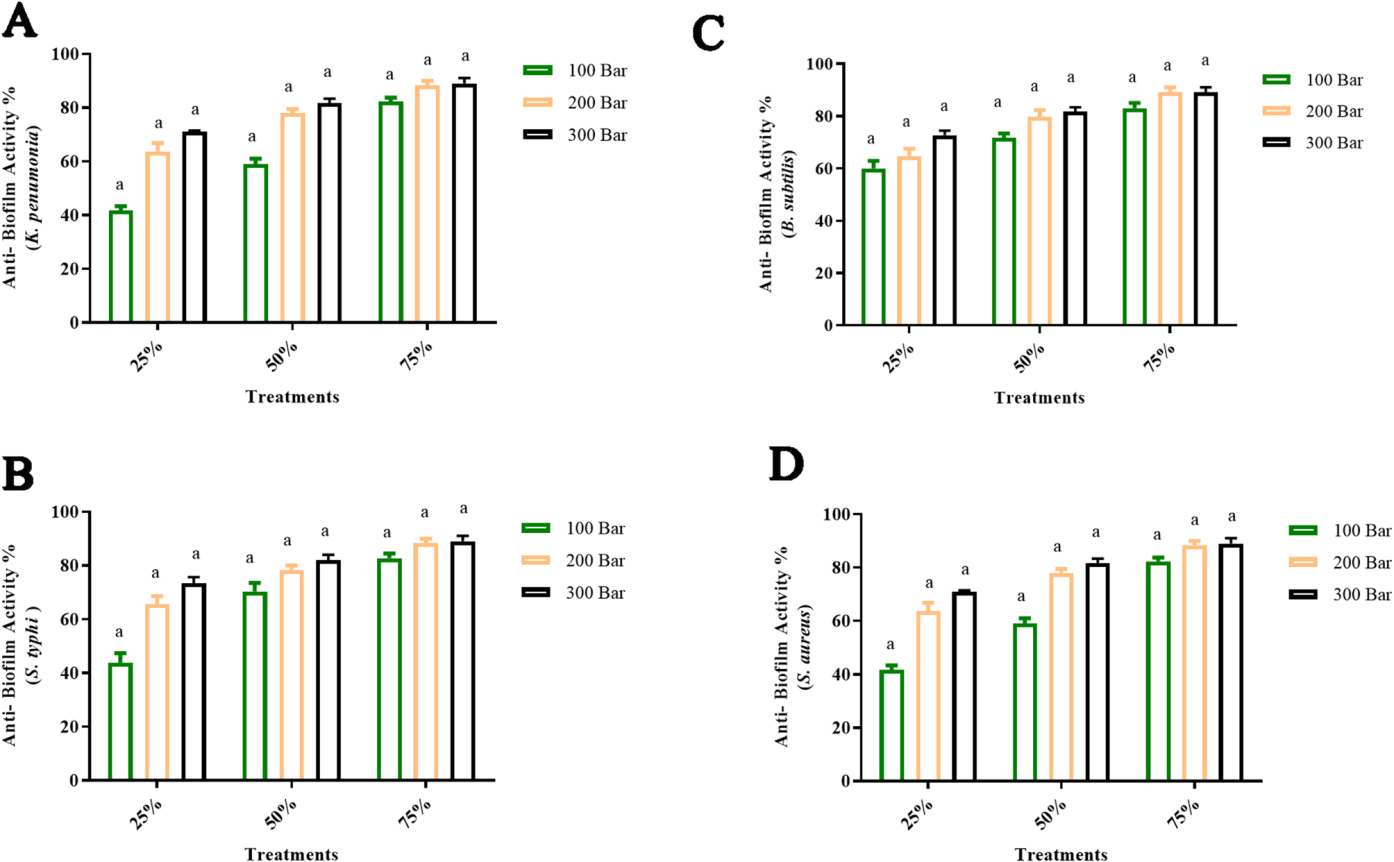
Statistical analysis for antibiofilm action of green cardamom extracts upon using various pressure levels (100, 200, and 300 bar) towards **A** *K. pneumonia,* **B** *S. typhi,* **C** *B. subtilis,* and **D** *S. aureus* outcomes are represnted as means ± SD). There is a notable change upon usig various extracts prearared upon application of various pressure levles with a non significant differnce *P* ≥ 0.05 as referd by simmilar letters above coulmns

Antibiofilm activity of the extract was estimated against tested bacteria as depicted in (Fig. 5). The biofilm formation was inhibited by the extract and raised with the increasing the extract concentration with a non-significant difference (*P* ≥ 0.05). At the same time the inhibition of biofilm formation was more affected by the extract at the extraction condition at 300 bar than that by the extract at the extraction condition at 100 and 200. The biofilm of *B. subtilis* was the most sensitive followed by *S. typhi, K. pneumonia,* and *S. aureus* where biofilm inhibition was 95.64 ± 0.98, 94.00 ± 1.22, 92.96 ± 0.66, and 92.32 ± 1.33%, respectively by the extract at extraction condition of 300 bar (Fig. 6). A bacterial biofilm is a multidimensional framework made up of mono- or multi-microbial populations attached to an outer layer of cells that gives microorganisms resistance to challenging circumstances, antimicrobial agents, and host immune response (Techaoei 2022).

The antibiofilm action of natural extracts can be strongly impacted by variations in their phenolic content. Improved antibacterial and antibiofilm qualities are frequently linked to higher concentrations of phenolic substances, such as flavonoids and phenolic acids. By disrupting with quorum detection, cell attachment, or by damaging bacterial cells, these substances can prevent the emergence and spread of biofilms (Bouchelaghem et al. 2022; Alejo-Armijo et al. 2025). Furthermore, Nassar et al. (2021) observed that microorganisms that cause a variety of human diseases and infections acquired in hospitals have demonstrated a biofilm origin. Accordingly, safe antibacterial substances that can hinder biofilm formation are needed to eradicate long-lasting infections linked to biofilms and lessen their detrimental effect on the health of people. The present investigation revealed that the increase in bioactive compounds upon elevation of the applied pressure enhanced the antibiofilm activity of green cardamom. Studies on the antibiofilm properties of shrub-derived phenolics have shown that, in addition to their harmful effects on bacteria, these compounds also have "softer" effects that minimize biofilms by interfering with bacterial regulating mechanisms like quorum recognition or other universal regulatory frameworks, all without influencing bacterial growth (Pourkhosravani et al. 2021). Generally, the remarked enhancement in antimicrobial and antibiofilm activity, along with the decrease in MIC and MBC of green cardamom extract with increasing extraction pressure, especially under SFE, can be attributed to several interrelated scientific reasons, where supercritical CO_2_ extraction becomes more efficient at higher pressures, enabling the solubilization and extraction of a greater concentration and wider range of bioactive compounds, such as: phenolics and flavonoids (Silva et al. 2017). Moreover, high pressures may extract a broader spectrum of compounds that can act synergistically, boosting the antimicrobial efficacy and biofilm disruption ability of the extract. Synergy among multiple phytochemicals can lead to lower MIC and MBC values, indicating stronger antimicrobial action at lower doses.

**Fig. 7:**
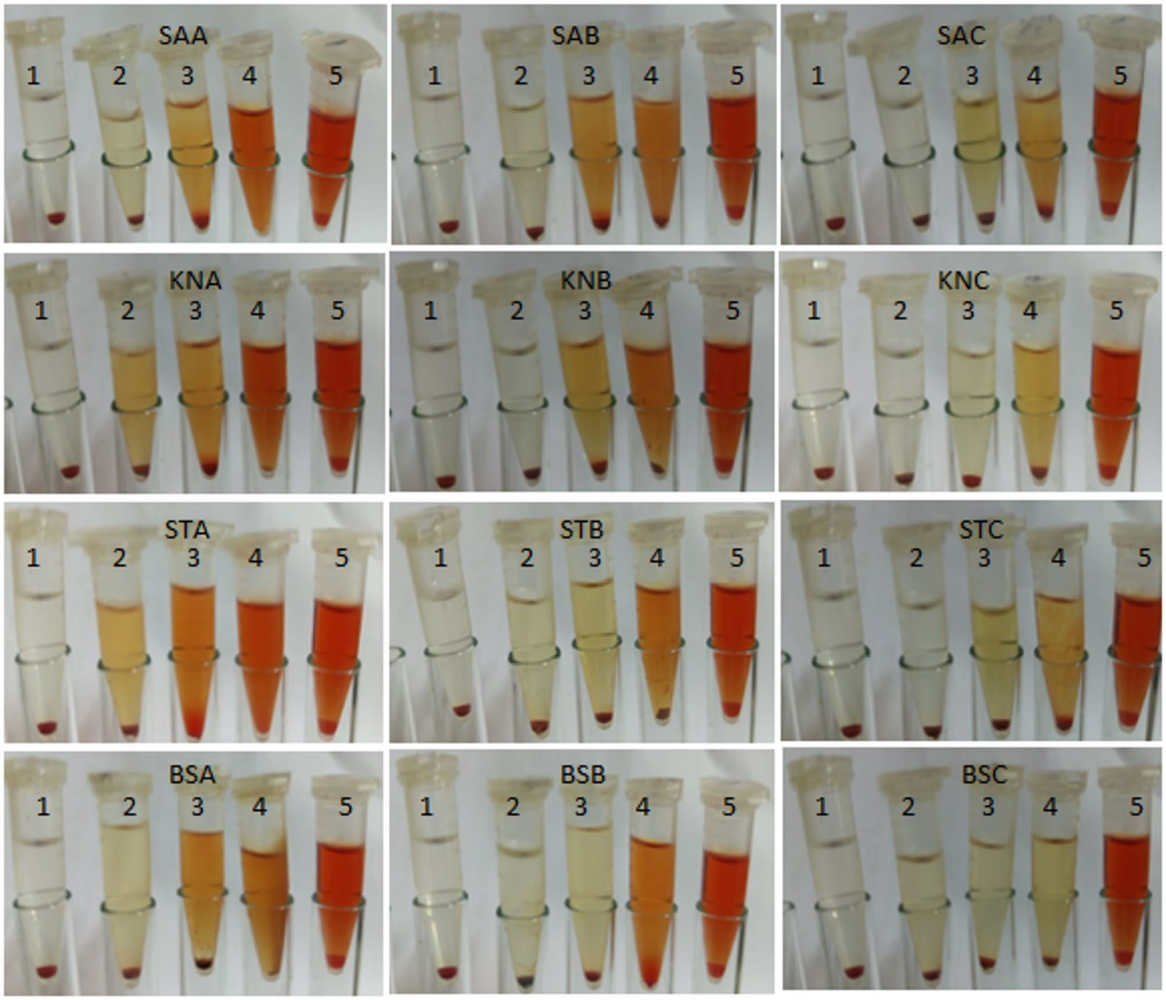
Hemolysis inhibition %. SAA *S. aureus* at 100 bar, SAB *S. aureus* at 200 bar, and SAC *S. aureus* at 300 bar. KNA *K. pneumonia* at 100 pressure, KNB *K. pneumonia* at 200 bar, and KNC *K. pneumonia* at 300 bar. STA *S. typhi at* 100 bar, STB *S. typhi* at 200 pressure, and STC *S. typhi* at 300 bar. BSA *B. subtilis* at 100 bar, BS *B. subtilis* at 200 pressure, and BS *B. subtilis* at 300 bar

**Fig. 8:**
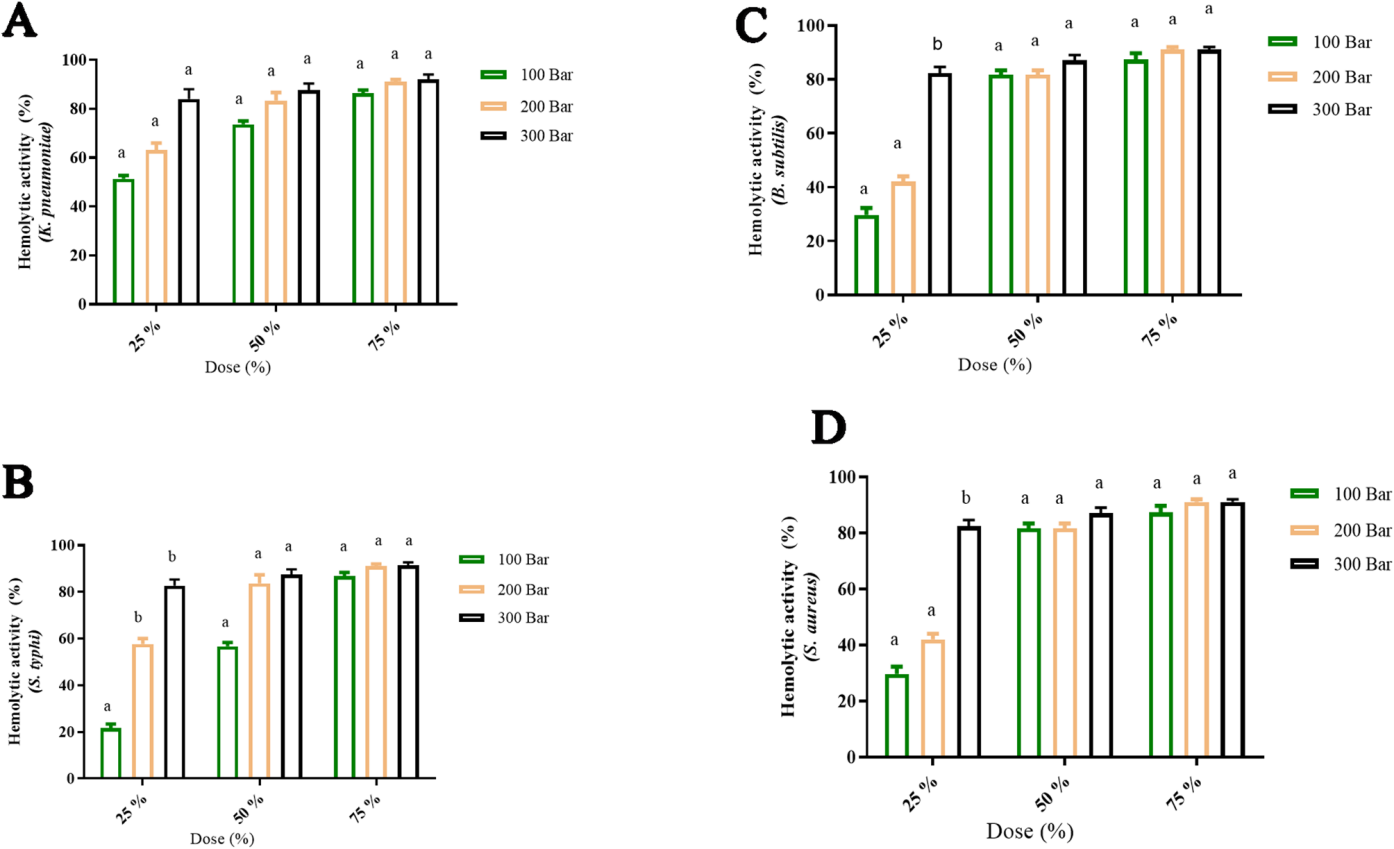
Statistical analysis for hemolysis inhibition % of green cardamom extracts upon using various pressure levels (100, 200, and 300 bar) in presence of **A*** K. pneumonia,* **B** *S. typhi,* **C** *B. subtilis,* and **D** *S. aureus* outcomes are represnted as means ± SD. There is a notable change upon usig various extracts prearared upon application of various pressure levles with where similar letters above coulms refers to significnat differnce *P* ≤ 0.05; while similar letters above colums refers to non signigficant differnce *P* ≥ 0.05)

The ability of tested extract to inhibite the hemolysis in the presence of tested bacteria was measured (Figs. 7, 8). As the concentration of extract increase, the hemolysis inhibition % increased as well as the extract at extraction condition 300 was effective for hemolysis inhibition % compared to the extract at extraction condition 200 and 100 bar in all cases of tested bacteria. At 75% MIC of the extract at extraction conditions 100, 200, and 300 bar, the hemolysis inhibition was 92.53 ± 2.41, 93.53 ± 1.10, and 94.27 ± 0.46% in the presence of *S. aureus,* 90.60 ± 3.11, 93.67 ± 1.76, and 94.80 ± 0.17% in the presence of *S. typhi,* 89.37 ± 0.47, 93.53 ± 3.41*,* and 96.66 ± 2.52% in the presence of *B. subtilis,* 90.33 ± 1.53, 93.70 ± 1.00, and 97.66 ± 2.08%, respectively in the presence of *K. pneumonia.* Since hemolysin is the primary feature of *S. aureus* strains’ virulence, it has been suggested that antivirulence/antitoxin strategies ought to prioritize it (Al-Rajhi and Abdelghany 2023a). The presnt result were in accordnace with research demenstrated that flavonoids have the capacity to aggressively counteract the hemolytic properties of *S. aureus*’s α-hemolysin, disrupting the structure of the toxin (Qanash et al. 2023a). An other way natural extracts exhibits antihemolytic effect is by strengthening the erythrocyte membrane’s resistance to the toxin (Qanash et al. 2023a).

Variations in phenolic content can have a major effect on a the prepared extracts antihemolytic actions, or thier capacity to stop or lessen hemolysis, the breakdown of red blood cells (erythrocytes). Increased antihemolytic action is typically linked to higher phenolic content because of their antioxidant qualities, which shield cells from oxidative harm that can cause hemolysis (Binsaleh et al. 2025).

Various types of green cardamom extracts which prepared upon using different pressure values (100, 200, and 300 bar) towards different examined bacteria at different time points could be seen at (Table 4). A dramatic reduction (*P* ≤ 0.05) in bacterial count could be seen upon using green cardamom extract which prepared at 300 bar relative to the other two forms of green cardamom extracts relative to control revealing its successive antimicrobial capacity at various tested time points which increased by various tested time intervals. The observed bacterial count indicated can be attributed to differences in the concentration and composition of bioactive compounds, such as phenolics and flavonoids of green cardamom extracts at different pressures—particularly the pronounced effect at 300 bar. These compounds are known for their antimicrobial effects, and their increased presence correlates with the significant reduction in bacterial count. A detailed analysis linking chemical composition to antibacterial activity would clarify which compounds are most responsible and highlight the impact of extraction pressure on bioactive yield. The decline in bacterial count in our investigation may be due to chlorogenic acid, Vanillin, and *p*-coumaric acid as mentioned in other studies (Makade et al. 2024; Stebuliauskaitė et al. 2025; Appiah et al. 2017). The prepared cardamom extract at 100 bar showed a minimal impact towards *B. subtilis* at different time points where 324 ± 12 bacterial cells could be detected after 180 min. There is no bacterial cells after exposing various bacteria for cardamom extract at 300 bar after 150 min revealing its effective impact at earlier examined time points. There is a marked increase (*P* ≤ 0.05) in the killing impact over time as well as a proportional elevation (*P* ≤ 0.05) with a notable difference upon elevation of the applied pressure for extraction (Table 4). Time killing kinetics confirmed the bactericidal impact of cardamom extract especially at the prepared one at 300 bar. A few research investigations have been conducted on the time-kill kinetics of cardamom, while numerous studies of natural product extracts have been published (Techaoei 2022). Killing examination revealed that the level of time-varying inhibition of microbes differed between bacteria and natural product extracts (Bouchelaghem et al. 2022). Plant-derived compounds and reactions following microbial infection may be taken into consideration when evaluating the antibacterial attributes (Alejo-Armijo et al. 2025).

**Table 4:** Effect of different Killing Kinetic times of green cardamom extracts upon using various pressure levels (100, 200, and 300 bar) towards *B. subtilis*, *S. aureus*, *K. pneumonia,* and *S. typhi,* outcomes are represnted as means ± SD)

Killing Kinetic time (min)	Control	CFU* at various pressure (bar)					
		100	200	300	100	200	300
		*B. subtilis*			*S. aureus*		
0	24 × 10^5^ ± 7^a^	26 × 10^5^ ± 12^a^	26 × 10^5^ ± 9 ^a^	26 × 10^5^ ± 2.0^a^	29 × 10^5^ ± 11^a^	29 × 10^5^ ± 12^a^	29 × 10^5^ ± 8^a^
30	8 × 10^4^ ± 4^b^	6 × 10^5^ ± 7^a^	56 × 10^4^ ± 6^b^	287 × 10^3^ ± 1^c^	232 × 10^4^ ± 9^a^	22 × 10^4^ ± 4^b^	15 × 10^4^ ± 6^b^
60	22 × 10^2^ ± 3^b^	128 × 10^4^ ± 8^a^	28 × 10^3^ ± 11^b^	18 × 10^2^ ± 2^b^	117 × 10^3^ ± 7^a^	79 × 10^3^ ± 8^a^	23 × 10^2^ ± 4^b^
120	150 ± 2^a^	184 × 10^3^ ± 11^a^	174 × 10 ± 4^a^	70 × 10 ± 3^b^	13 × 10^2^ ± 6^a^	210 ± 6^b^	167 ± 3^a^
150	0^c^	25 × 10^2^ ± 12^a^	291 ± 3^b^	0^c^	380^a^	21 ± 2^b^	0^c^
180	0^a^	324 ± 12^a^	0^b^	0^b^	0^a^	0^a^	0^a^
Killing Kinetic time (min)	Control	*K. pneumoniae*			*S. typhi*		
0	5 × 10^5^ ± 3^a^	24 × 10^5^ ± 8^a^	24 × 10^5^ ± 12^a^	24 × 10^5^ ± 9^a^	5 × 10^5^ ± 6^a^	5 × 10^5^ ± 1^a^	5 × 10^5^ ± 4^a^
30	18 × 10^4^ ± 9^c^	239 × 10^4^ ± 6^a^	30 × 10^4^ ± 14^b^	13 × 10^4^ ± 8^c^	221 × 10^4^ ± 8^a^	94 × 10^4^ ± 2^b^	19 × 10^4^ ± 11^c^
60	11.8 × 10^3^ ± 14^c^	157 × 10^3^ ± 5^a^	17 × 10^3^ ± 8^b^	213 × 10^2^ ± 11^c^	242 × 10^3^ ± 4^a^	142 × 10^3^ ± 1^b^	12 × 10^3^ ± 14^c^
120	190 ± 7^c^	176 × 10 ± 4^a^	250 ± 7^b^	231^b^	126 × 10^2^ ± 6^a^	15 × 10^2^ ± 2^b^	200 ± 8^c^
150	0^b^	193 ± 3^a^	11 ± 4^b^	0^c^	292^a^	294^a^	0^b^
180	0^a^	0^a^	0^a^	0^a^	0^a^	0^a^	0^a^

*The statistical comparison for different treatments upon using various pressure levels and control showed a significant variations where different letters a: b *P* ≤ 0.05; b: c *P* ≤ 0.01; a: c *P* ≤ 0.01 & N.B: there is a gradual significant increase (*P* ≤ 0.05 (in the impact of tested samples upon elevation of time points (0–180 min) for the same sample tested versus same microbe over time ( comparison among values in the same columns)

**Fig. 9:**
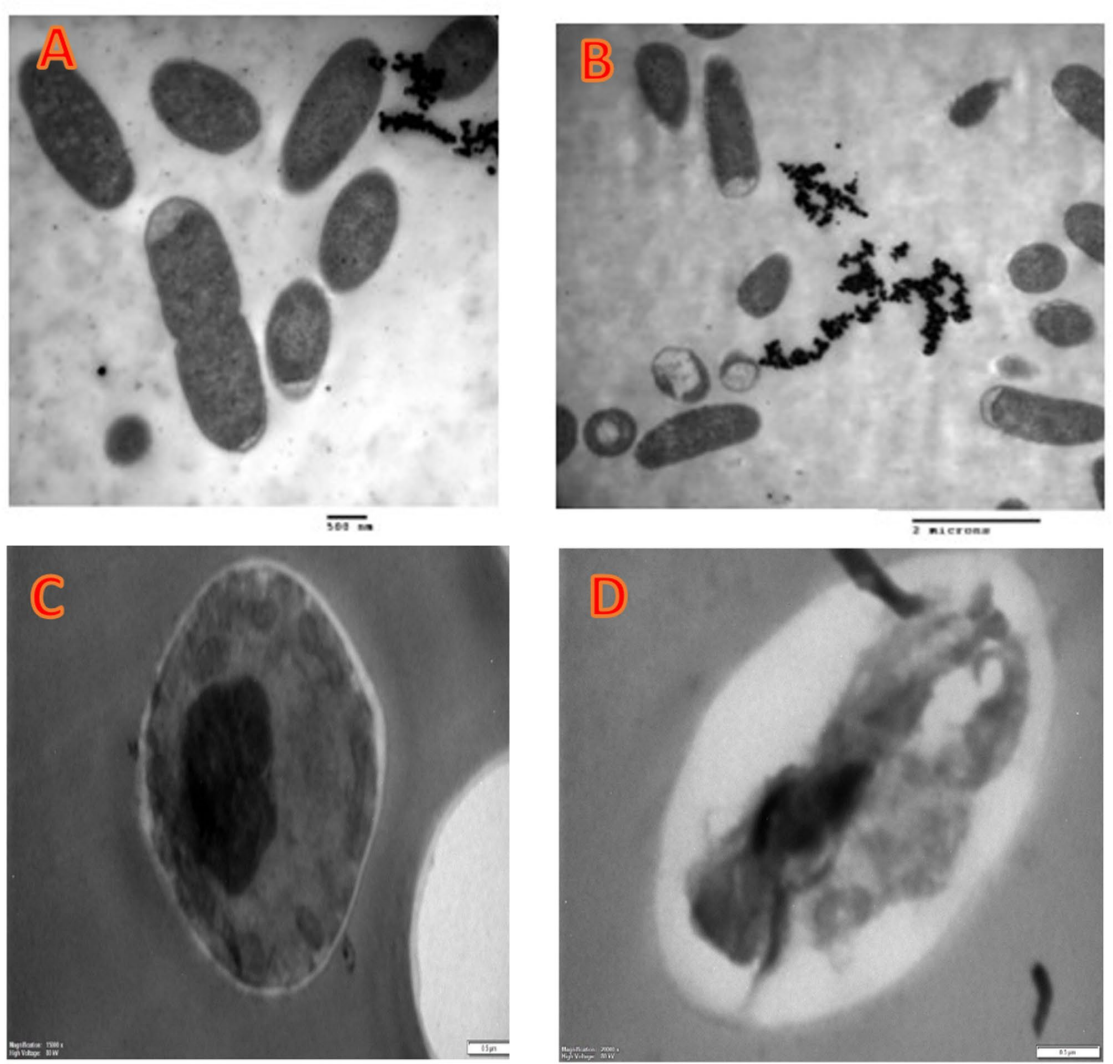
TEM micrographs for **A** *B. subtilis*, **B** *B. subtilis* treated by cardamom extract obtained by SFE extraction at 300 bar, **C** control * C. albicans,* and **D** *C. albicans* cells treated by cardamom extract obtained by SFE extraction at 300 bar (Magnification 10,000 X)

The green cardamom extract obtained by SFE extraction at 300 bar caused numerous ultrastructural changes in *B. subtilis* and *C. albicans* cells in the current investigation. TEM images (Fig. 9A) of the untreated *B. subtilis* specimen showed that each individual cell was healthy, rod-shaped, and encompassed by the outermost and innermost layers with no any damage to the cell. When *B. subtilis* cells were treated with green cardamom extract, their size drastically shrank and several lysed cells were visible (Fig. 9B). TEM pictures (Fig. 9C) showed that individual *C. albicans* cells in the reference specimen were well, rounded, and classically shaped; however, when green cardamom was applied, the *C. albicans* cells were destructed, resulting in deformed cells with a diminished shape (Fig. 9D). To fight the microbes that cause different infections and illnesses, new antimicrobial substances had to be created (Silva et al. 2017; Al-Rajhi and Abdelghany 2023a). The TEM technique can provide valuable insight into the antibacterial mechanisms of action of new antibacterial substances to confirm the antimicrobial outcomes through visualization of various alterations upon treatments (Qanash et al. 2023a).

### In silico investigation though docking analysis

Syringic acid showed stronger binding (S = − 4.27 to − 4.64 kcal/mol for 3V8J; S = − 4.69 to − 5.04 kcal/mol for 5VX6) than gallic acid (S = − 4.11 to − 4.45 kcal/mol for 3V8J; S = − 4.52 to − 4.68 for 5VX6). Lower RMSD_refine values indicated stable poses. E_conf values reflecting conformational stability. E_place and E_refine values were consistently favorable.

**Fig. 10.**
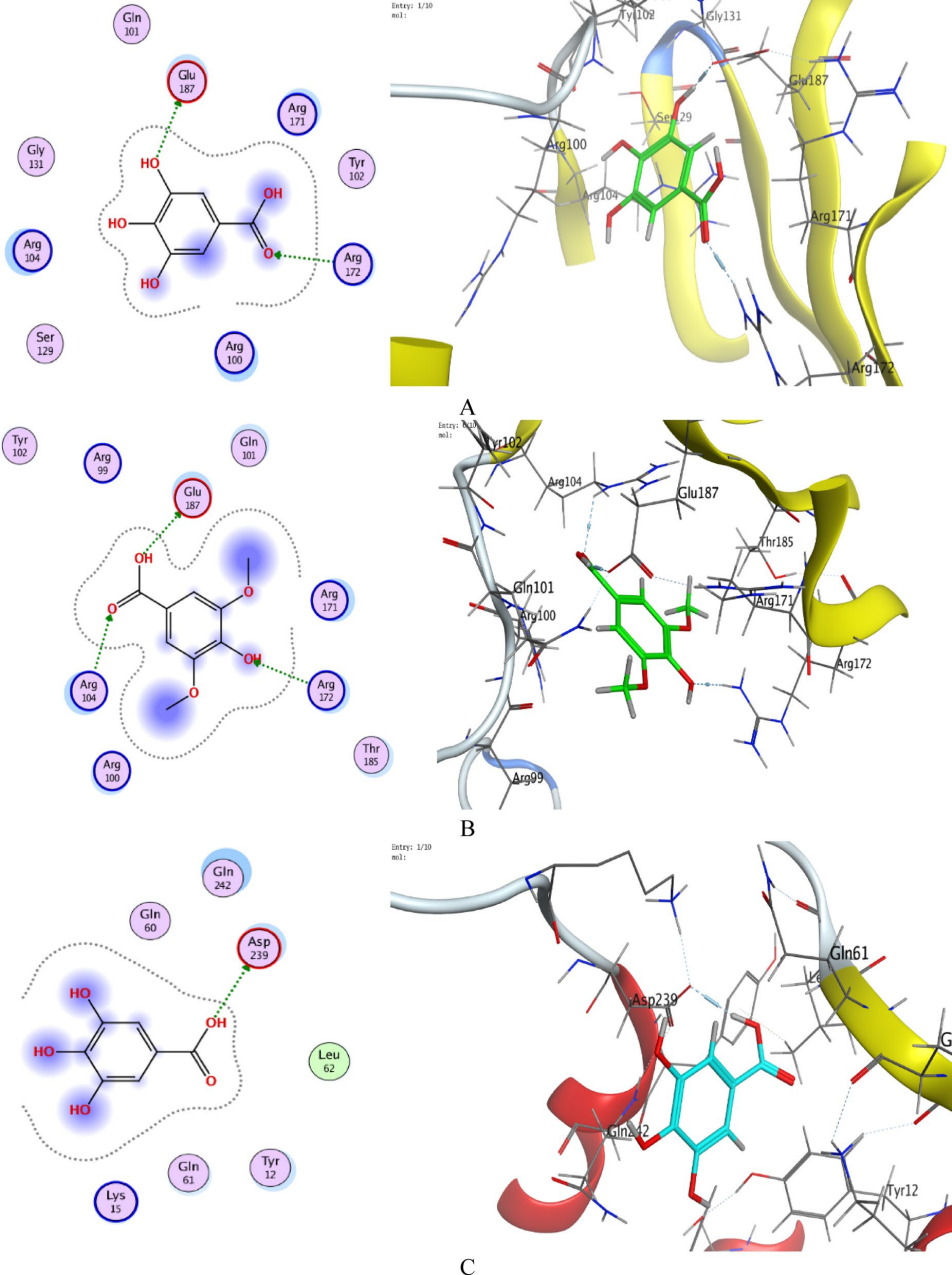

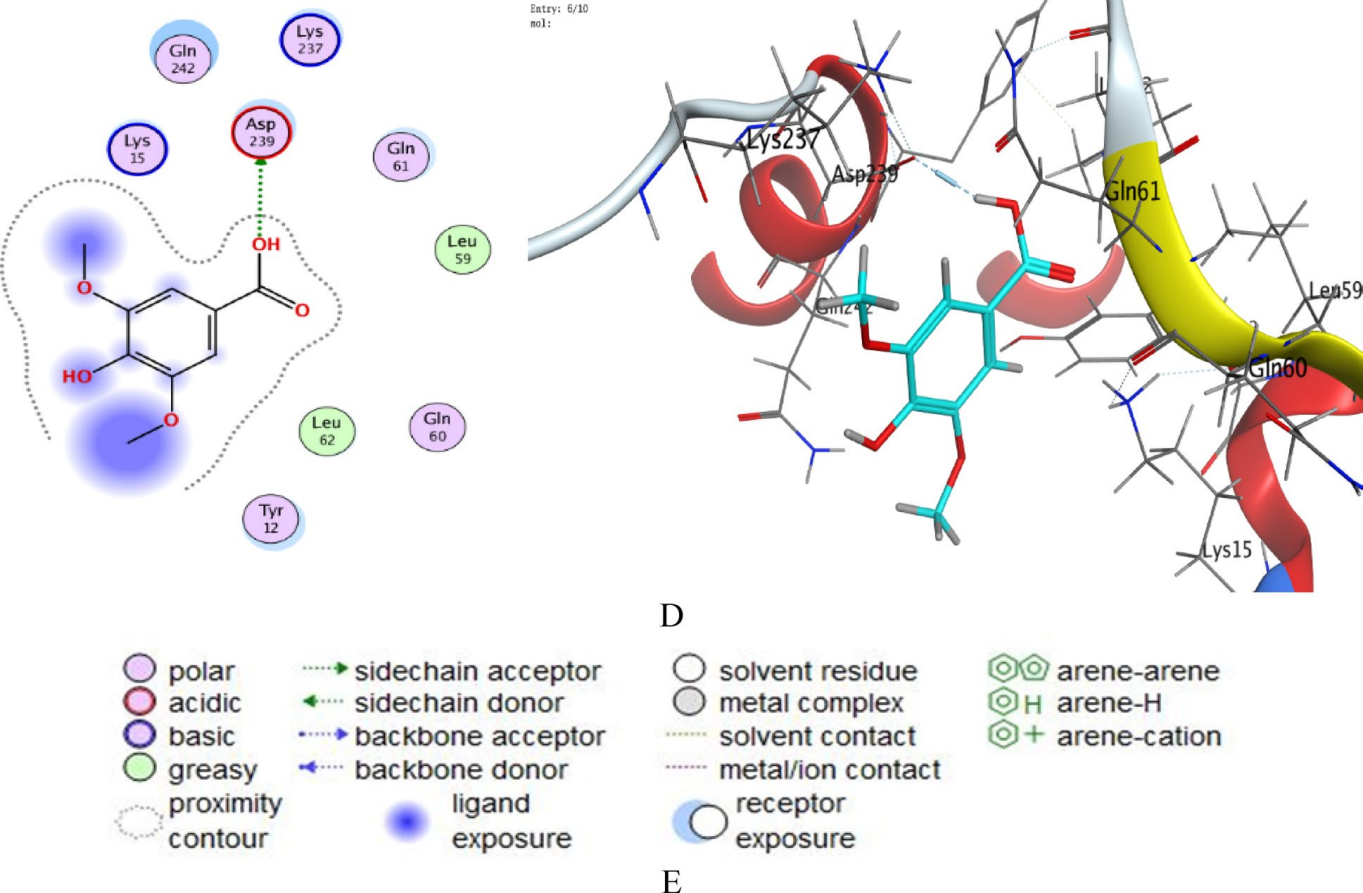
2D and 3D diagrams show the interaction between gallic acid and active sites of *B. subtilis* 5VX6 protein (**A**), 2D and 3D diagrams show the interaction between syringic acid and active sites of *B. subtilis* 5VX6 protein (**B**), 2D and 3D diagrams show the interaction between gallic acid and active sites of *S. aureus* 3V8J protein (**C**), 2D and 3D diagrams show the interaction between syringic acid and active sites of *S. aureus* 3V8J protein (**D**), and the representative key for the types of interaction between ligands and selected protein receptors (**E**)

Key Interactions for *B. subtilis* (5VX6) indicated that gallic acid formed H-bonds with GLU 187 (2.73 Å, − 5.4 kcal/mol) and ARG 172 (2.94 Å, − 4.4 kcal/mol). Syringic acid interacted with GLU 187 (2.82 Å, − 7.3 kcal/mol), ARG 172 (3.10 Å, − 1.4 kcal/mol), and AR 104 (3.04 Å, − 0.8 kcal/mol). In *S. aureus* (3V8J): Both acids bound to ASP 239 (Gallic acid: 2.85 Å, − 5.4 kcal/mol; Syringic acid: 2.86 Å, − 4.8 kcal/mol). The important docking metrics, such as docking scores, interaction types, binding energies, and distances, have been reviewed to determine binding affinities and interaction processes, as shown in (Tables 5, 6, 7 and 8). The best-fitted diagrams of 2D and 3D poses select by the syringic acid and gallic acid are described (Fig. 10). Regarding the discussion of docking analysis, the results reveal nuanced differences in the binding behavior of gallic acid and syringic acid against *B. subtilis* (5VX6) and *S. aureus* (3V8J), offering insights into their potential as antimicrobial agents. The presence of methoxy groups in syringic acid likely enhances its binding. While methoxy groups are not directly involved in hydrogen bonding, their electron-donating effects may subtly modulate the electronic environment of syringic acid’s hydroxyl groups, enhancing their ability to form stable bonds with residues like GLU 187. In *B. subtilis* (5VX6), syringic acid forms a stronger hydrogen bond with GLU 187 (− 7.3 kcal/mol) compared to Gallic acid (− 5.4 kcal/mol), likely due to optimal alignment of its hydroxyl groups. Additional interactions with ARG 172 and ARG 104 (Table 7) may stabilize its pose despite the absence of methoxy group participation.

**Table 5:** Docking scores and energies of gallic acid and syringic acid with structure of *B. subtilis* (PDB ID: 5VX6)

Mol	S	rmsd_refine	E_conf	E_place	E_score1	E_refine	E_score2
Gallic acid	− 4.67921	1.7332464	− 31.8259	− 56.9843	− 9.70131	− 23.0596	− 4.67921
Gallic acid	− 4.59409	0.74464136	− 32.8286	− 68.1666	− 10.2249	− 24.6589	− 4.59409
Gallic acid	− 4.58793	1.7696532	− 33.2433	− 63.2359	− 9.91201	− 25.4289	− 4.58793
Gallic acid	− 4.56066	2.6160221	− 31.7368	− 52.0508	− 10.3878	− 23.347	− 4.56066
Gallic acid	− 4.51797	2.1812108	− 33.1922	− 47.8645	− 9.88048	− 21.0348	− 4.51797
Syringic acid	− 5.03796	0.52116263	− 14.0667	− 78.0995	− 10.1364	− 28.6602	− 5.03796
Syringic acid	− 4.94237	0.94439775	− 13.9608	− 70.5491	− 10.0975	− 25.0605	− 4.94237
Syringic acid	− 4.92053	2.111726	− 5.60061	− 64.9127	− 10.0869	− 22.5365	− 4.92053
Syringic acid	− 4.69705	1.4523685	− 13.7555	− 67.6678	− 10.1653	− 22.6829	− 4.69705
Syringic acid	− 4.67621	1.2140613	− 7.97653	− 61.7188	− 10.9312	− 21.6721	− 4.67621

**Table 6:** Docking scores and energies of gallic acid and syringic acid with structure of *S. aureus* (PDB ID: 3V8J)

Mol	S	rmsd_refine	E_conf	E_place	E_score1	E_refine	E_score2
Gallic acid	− 4.45374	1.4267793	− 32.8569	− 50.573	− 9.60852	− 22.523	− 4.45374
Gallic acid	− 4.28895	1.3745345	− 30.6581	− 53.7743	− 9.88156	− 19.5394	− 4.28895
Gallic acid	− 4.19007	2.6599638	− 33.9026	− 41.8718	− 11.3473	− 17.8853	− 4.19007
Gallic acid	− 4.18197	1.3183807	− 32.5074	− 69.8018	− 11.9495	− 17.2698	− 4.18197
Gallic acid	− 4.11419	1.4595817	− 32.7511	− 55.7456	− 9.92507	− 15.8203	− 4.11419
Syringic acid	− 4.64437	0.73497051	− 12.4447	− 60.9532	− 10.6667	− 23.4391	− 4.64437
Syringic acid	− 4.52733	0.98655659	− 7.59219	− 61.9997	− 10.1858	− 19.1627	− 4.52733
Syringic acid	− 4.31496	1.6934763	− 10.5394	− 60.394	− 10.1252	− 19.3342	− 4.31496
Syringic acid	− 4.28199	1.5533125	− 10.5155	− 61.2405	− 9.95271	− 17.4618	− 4.28199
Syringic acid	− 4.2695	3.3460429	− 10.8598	− 58.6044	− 10.1436	− 17.6905	− 4.2695

**Table 7:** Interaction of gallic acid and syringic acid with structure of *B. subtilis* (PDB ID: 5VX6)

Mol	Ligand	Receptor	Interaction	Distance	E (kcal/mol)
Gallic acid	O 13	OE2 GLU 187 (A)	H-donor	2.73	− 5.4
	O 13	NH1 ARG 172 (A)	H-acceptor	2.94	− 4.4
Syringic acid	O 23	OE2 GLU 187 (A)	H-donor	2.82	− 7.3
	O 14	NH1 ARG 172 (A)	H-acceptor	3.10	− 1.4
	O 22	NE ARG 104 (A)	H-acceptor	3.04	− 0.8

**Table 8:** Interaction of gallic acid and syringic acid with structure of *S. aureus* (PDB ID: 3V8J)

Mol	Ligand	Receptor	Interaction	Distance	E (kcal/mol)
Gallic acid	O 17	OD2 ASP 239 (A)	H-donor	2.85	− 5.4
Syringic acid	O 23	OD2 ASP 239 (A)	H-donor	2.86	− 4.8

*Steric compatibility* Despite its larger size, Syringic acid’s structure appears better accommodated in the binding pockets, as reflected in lower RMSD_refine values (e.g., 0.52 Å in 5VX6), indicating stable pose retention post-refinement.

*Energy term analysis* indicated that gallic acid showed more favorable conformational energy (E_conf) values (− 31.8 to − 33.9 kcal/mol vs. Syringic acid’s − 5.6 to − 14.1 kcal/mol), suggesting its simpler structure adopts lower-energy conformations. However, syringic acid’s higher (less negative) E_conf may reflect conformational adjustments to maximize interactions, ultimately yielding stronger overall binding (S).

*Placement and refinement energies: *Syringic acid’s superior E_place (e.g., − 78.10 kcal/mol in 5VX6) indicates efficient initial docking, while its moderate E_refine penalties (e.g., − 28.66 kcal/mol) suggest minor adjustments to optimize interactions. Several investigators employed the molecular docking to document the activity of some phenolic and flavonoids against cancer cells, pathogenic bacteria, and yeasts (Famuyide et al. 2020; Al-Rajhi and Abdelghany 2023b; Qanash et al. 2023b, c). For example, catechin and gallic acid were docked with prostate cancer cells 3 PDB ID: 2Q7L with binding affinities − 5.27521 and − 6.3204 kcal/mol (Binsaleh et al. 2025). Rosmarinic acid was docked with *C. tropicalis* (PDB ID: 6ZD6), *G. candidum* (PDB ID: 6ISV), and *C. albicans* (PDB ID: 1ZAP) with binding affinities − 6.8224 kcal/mol, − 6.79996, and − 6.15839 kcal/mol, respectively (Bazaid et al. 2025). According to Alsalamah et al. (Alsalamah et al. 2023), ellagic acid showed − 4.5145 kcal/mol and − 6.18615 kcal/mol, while chlorogenic acid showed − 5.69876 kcal/mol and − 7.84379 kcal/mol as docked with *G. candidum* (4ZZT) and *C. albicans* (4ZZT) proteins, respectively.

## Conclusion

This is a novel investigation to extract a substantial quantity of phenolic components from green cardamom employing the SFE approach. The SFE apparatus’s pressure has been adjusted to maximize both the yield and the quality of the extracted substances. Remarkably, in contrast to the other conditions of treatment, the extracted output rose under high pressure (300 bar). Additionally, a large number of chemicals in extracted green cardamom have been found to have about 13 bioactive components using the HPLC method. Raising of the pressure from 100 to 300 alter the levels of the extracted compounds with a significant raise in gallic acid. The detected compounds are recognized to have both medicinal and effective antimicrobial properties and improve the pattern for time killing testing towards test organisms relative to control. The best results for antibiofilm as well as hemolysis inhibition could be detected by the prepared extract at 300 bar relative to the other two extracts prepared at lower pressure levels. Furthermore, the existence of Flavonoids and the phenolic compounds in appropriate quantities in the used cardamom extract can increase antimicrobial activity. Their capacity to interact with and damage different bacterial cellular targets, including cell membranes, and obstruct essential microbial functions like energy metabolism and DNA replication, as demonstrated by ultrastructure alteration tested by electron microscopy, as the reason for this improvement. Syringic and gallic acids exhibit promising binding to *B. subtilis* and *S. aureus* targets, with syringic acid showing superior affinity. The interactions with key catalytic residues (GLU 187, ASP 239) provide a mechanistic basis for inhibition. Combining both in vitro and in silico results give a proper direction for the effectiveness of application of optimal pressure level for enhancing beneficial impact of green cardamom to be applied in animal experimental models for future applications.

## Data Availability

The results from the current investigation are available from the corresponding author upon reasonable appeal.

## Abbreviations

SFE: Supercritical fluid extraction
PDB: Protein data bank
MOE: Molecular operating environment
RMSD_refine: Root mean square deviation refined
E_conf: Conformational energy
ROS: Reactive oxygen species
HPLC: High-performance liquid chromatography
PDA: Photodiode array
TFA: Trifluoroacetic acid
UV–Vis: Ultraviolet–visible spectroscopy
ATCC: American type culture collection
MIC: Minimal inhibitory concentration
MBC: Minimal bactericidal concentration
CFU: Colony-forming unit
TSYB: Trypticase soy yeast broth
TEM: Transmission electron microscope
SD: Standard deviation
GLU 187: Glutamic acid residue at position 187 in the protein sequence
ARG 172: Arginine residue at position 172
ASP 239: Aspartic acid residue at position 239
5VX6: A crystal structure deposited in the PDB
3V8J: Another crystal structure in the PDB
